# Preparation of metal phthalocyanine (MPc)–polymer complexes: the possible anti-cancer properties of FePc–polymer complexes

**DOI:** 10.1016/j.heliyon.2019.e01383

**Published:** 2019-03-29

**Authors:** Isamu Inamura, Kentaro Inamura, Yuji Jinbo, Toshinao Mihara, Yasuhiro Sasaoka

**Affiliations:** a Department of Materials Science, Faculty of Science and Engineering, Shimane University, Matsue 690-8504, Japan; b Division of Pathology, The Cancer Institute, Japanese Foundation for Cancer Research, Tokyo 135-8550, Japan; c Department of Biochemical Engineering, Graduate School of Science and Engineering, Yamagata University, Yonezawa 992-8510, Japan

**Keywords:** Materials science, Materials chemistry, Cancer research, Pharmaceutical chemistry

## Abstract

We have succeeded in preparing various water-soluble metal phthalocyanine (MPc)–polymer complexes, wherein the metal moiety is lithium, iron, cobalt, copper, zinc, or tin, and the polymer is one of the following water-soluble polymers: polyethylene glycol (PEG), polyvinyl pyrrolidone (PVP), or polyvinyl alcohol (PVA). Among all MPc–polymer complexes, the iron phthalocyanine (FePc)–PVP complex in water showed the largest and sharpest absorption peak at ∼700 nm in UV–Vis absorption spectrum, which indicates that FePc–polymer complexes in water are easily prepared and the degree of stacking of FePc in the complexes, very small, such as that of a monomer or a similar structure. Conversely, the polymer chains including those of PEG, PVP, and dextran have high biological affinity as well as flexibility. Speculatively, the FePc–polymer (e.g., PEG, PVP, and dextran) complexes adsorbed onto the surface of a cancer cell might break it via the irradiation of near-infrared light having a wavelength of ∼700 nm. Furthermore, chlorophyll a–polymer complexes, previously prepared by our group, might similarly break a cancer cell because these complexes showed a large and sharp absorption peak at ∼700 nm in UV–Vis spectrum.

## 1 Introduction

As known well, metal phthalocyanines (MPcs) and chlorophyll a are hydrophobic substances and therefore insoluble in water. In previous studies, we were successful in preparing the water-soluble chlorophyll a–polymer complexes by using the water-soluble polymers such as polyethylene glycol (PEG), polyvinyl pyrrolidone (PVP), dextran, or polyvinyl alcohol (PVA) [1, 2, 3, 4, 5, 6]. The typical method for preparing the complexes involves the initial preparation of a solution comprising chlorophyll a, a polymer, and an organic solvent [3, 4]. In particular, the organic solvents utilized for this purpose were methanol, ethanol, acetone, or petroleum ether, for which the boiling temperature (T_b_) is relatively low. However, since MPcs are insoluble in these organic solvents, water-soluble MPc–polymer complexes cannot be prepared using this route and these organic solvents.

Alternatively, it is well known that MPcs dissolve in organic solvents with a very high value of T_b_, such as pyridine, dimethyl sulfoxide (DMSO), N,N-dimethylformamide (DMF), or N,N-dimethylacetamide (DMA). Using these organic solvents, we successfully prepared the various water-soluble MPc–polymer complexes. The water-soluble MPc–polymer complexes obtained probably have the potential of being used in numerous fields.

Recently, H. Kobayashi *et al.* [7, 8, 9] succeeded in breaking a cancer cell and a Treg-cell by irradiating a phthalocyanine (IR700)–antibody conjugate bound to the surface of these cells with near-infrared light, i.e., light having a wavelength of ∼700 nm. Here the antibody in the conjugate has the ability to bind to the surface of these cells. This new method is called near-infrared photoimmunotherapy (NIR-PIT). In the case of NIR-PIT, both the cancer cell and the Treg-cell are directly broken by the heat generated from a phthalocyanine (IR700).

Speculatively, FePc–polymer (e.g., PEG, PVP, and dextran) complexes adsorbed onto the surface of a cancer cell might similarly break the cancer cell via irradiation process of the near-infrared light having a wavelength of ∼700 nm because, in the present study, the FePc–PVP complex in water exhibited the largest and sharpest absorption peak at ∼700 nm in the UV–Vis absorption spectrum among all the MPc–polymer complexes. Herein, it is considered that the largest and sharpest absorption peak indicates that FePc–polymer complex can be easily formed, and thus can be easily prepared, and the degree of stacking of FePc in the complexes is very small, such as that of a monomer or a similar structure. Similarly, chlorophyll a–polymer (e.g., PEG, PVP, and dextran) complexes might break cancer cells via irradiation of near-infrared light because the complexes in water showed a large and sharp absorption peak at ∼700 nm [1, 2, 3, 4, 5, 6]. Although NIR-PIT requires expensive antibodies, our method requires no antibodies by using the most suitable polymer chain.

## 2 Materials and methods

### 2.1 Chemicals

**Fig. 1:**
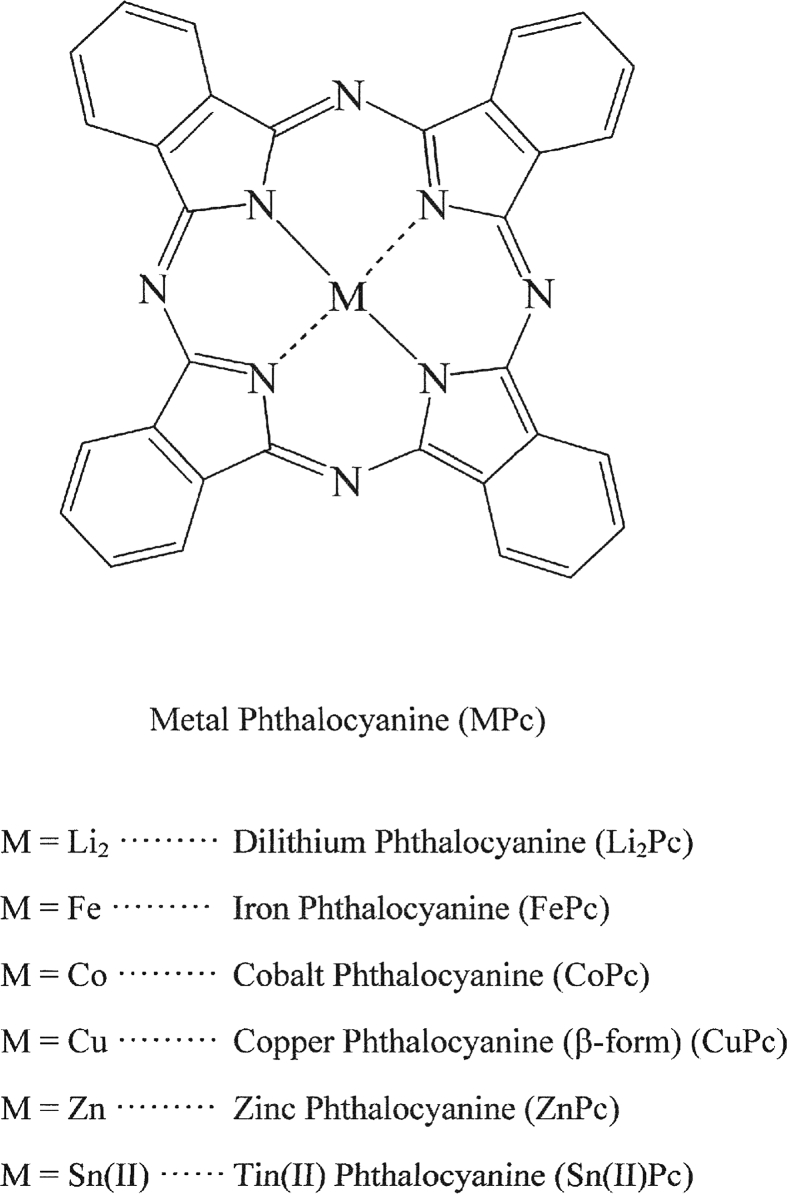
Chemical structure of a metal phthalocyanine and abbreviations referring to various similar species.

As shown in Fig. 1, MPcs contain Li_2_Pc, FePc, CoPc, CuPc (β-form), ZnPc, and SnPc. These MPc samples were purchased from Tokyo Chemical Industry Co., Ltd. and were used without further purification. Since MPcs have a conjugated double bond system, these species tend to stack with each other by way of π–π interaction.

PEG (average molecular weight 20,000) and PVP (average molecular weight 40,000) were purchased from Kishida Chemical Co., Ltd. PVA (average molecular weight 24,000 and degree of saponification 98.5 mol%) was donated from Kuraray Co., Ltd. Notably, PVA can be dissolved in water at a temperature higher than 95 °C.

Pyridine (T_b_ = 116 °C), DMSO (T_b_ = 189 °C), DMF (T_b_ = 153 °C), and DMA (T_b_ = 166 °C (754 mmH_g_)) were purchased in JIS special grade (purity ≥99%) from Kanto Chemical Co., Inc. and used without further purification.

Ion-exchanged water was purchased from Kishida Chemical Co., Ltd. and used without further purification.

### 2.2 Protocol for preparing the water-soluble MPc–polymer complexes

Step (1) MPc (0.01 g), polymer (PEG, PVP, or PVA) (1 g), and organic solvent (pyridine, DMSO, DMF, or DMA) (5 g) were mixed in a beaker, and the resulting mixture was gently stirred with a micro-spatula at 40 °C (for PEG and PVP) or at 95 °C (for PVA), until a homogeneous paste was formed. At this stage, the molar ratio of polymer/MPc is 3.0 in the case of PEG, 1.5 in the case of PVP, and 2.4 in the case of PVA. As an example, we explain the method for calculating the molar ratio of PEG/MPc as follows:
  molecular weight: Li_2_Pc = 526 (minimum)
  ∴ Li_2_Pc (0.01 g) = 0.01/526 mol = 1.9 × 10^−5^ mol
  molecular weight: Sn(II)Pc = 631 (maximum)
  ∴ Sn(II)Pc (0.01 g) = 0.01/631 mol = 1.6 × 10^−5^ mol
  molecular weight: PEG = 20,000 ∴ PEG (1 g) = 1/20,000 mol = 5 × 10^−5^ mol
  molar ratio: PEG/Li_2_Pc = 5 × 10^−5^/1.9 × 10^−5^ = 2.6
  molar ratio: PEG/Sn(II)Pc = 5 × 10^−5^/1.6 × 10^−5^ = 3.1
  ∴ molar ratio: PEG/MPc = 2.6–3.1 ∴ molar ratio: PEG/MPc = 3.0
Step (2) 2 g of the paste was put into a test tube. The test tube was then placed in a silicone oil bath kept at a constant temperature (10–20 °C below the T_b_ of the organic solvent).
Step (3) The paste was then dried under a reduced pressure (1.7 kPa) using an aspirator. At this time, the paste was quickly transformed to a porous solid material consisting of MPc and polymer.
Step (4) The temperature of the silicone oil bath was reduced to 40 °C (in the cases of PEG and PVP) and 95 °C (in the case of PVA). Subsequently, air was allowed to flow into the test tube until the pressure on the test tube reached the atmospheric pressure.
Step (5) A small amount of water was then added to the solid present in the test tube, and the resulting mixture was gently stirred with a micro-spatula at 40 °C (in the case of the mixture containing PEG or PVP) and 95 °C (in the case of the mixture containing PVA) until a homogeneous paste consisting of MPc, polymer, and water was obtained.
Step (6) The paste in the test tube was diluted with water to a given concentration and the test tube was removed from the silicone oil bath. As a result, a mixture comprising a water-soluble MPc–polymer complex was obtained at room temperature.
Step (7) The mixture thus obtained was filtered through a nitrocellulose-type membrane filter having a pore size of 0.45 μm. At this point, an aqueous solution of the MPc–polymer complex was obtained successfully. The color of this solution was blue for all the complexes that were synthesized.
Note (1) The porous solid material obtained in Step (3) may still contain a small amount of organic solvent molecules owing to a very high value of T_b_ of the solvents utilized in this study. Therefore, the aqueous solution of the MPc–polymer complex obtained toward the end of Step (7) may still contain small amounts of the organic solvent. In fact, when pyridine was used as the organic solvent, the filtered solution obtained in Step (7) smelled like pyridine. However, using a strong vacuum pump at Step (3), the organic solvent molecules will be removed almost completely. Furthermore, it is strongly expected that almost all the organic solvent molecules will be removed by the dialysis membrane method after Step 7.
Note (2) Pyridine is strongly recommended as an organic solvent to dissolve MPc because the value of T_b_ for pyridine is the lowest among used organic solvents and the presence of even a small amount of pyridine in the MPc–polymer complex aqueous solution can be easily detected by smell.

### 2.3 Measurement of UV–Vis absorption spectra

UV–Vis absorption spectra of the MPc–polymer complexes in water and those of MPcs in organic solvents were measured using a double beam spectrophotometer, UVIDEC-510 (Japan Spectroscopic Co., Ltd.), at room temperature.

## 3 Results and discussion

### 3.1 UV–Vis absorption spectra of MPc–polymer complexes in water

**Fig. 2:**
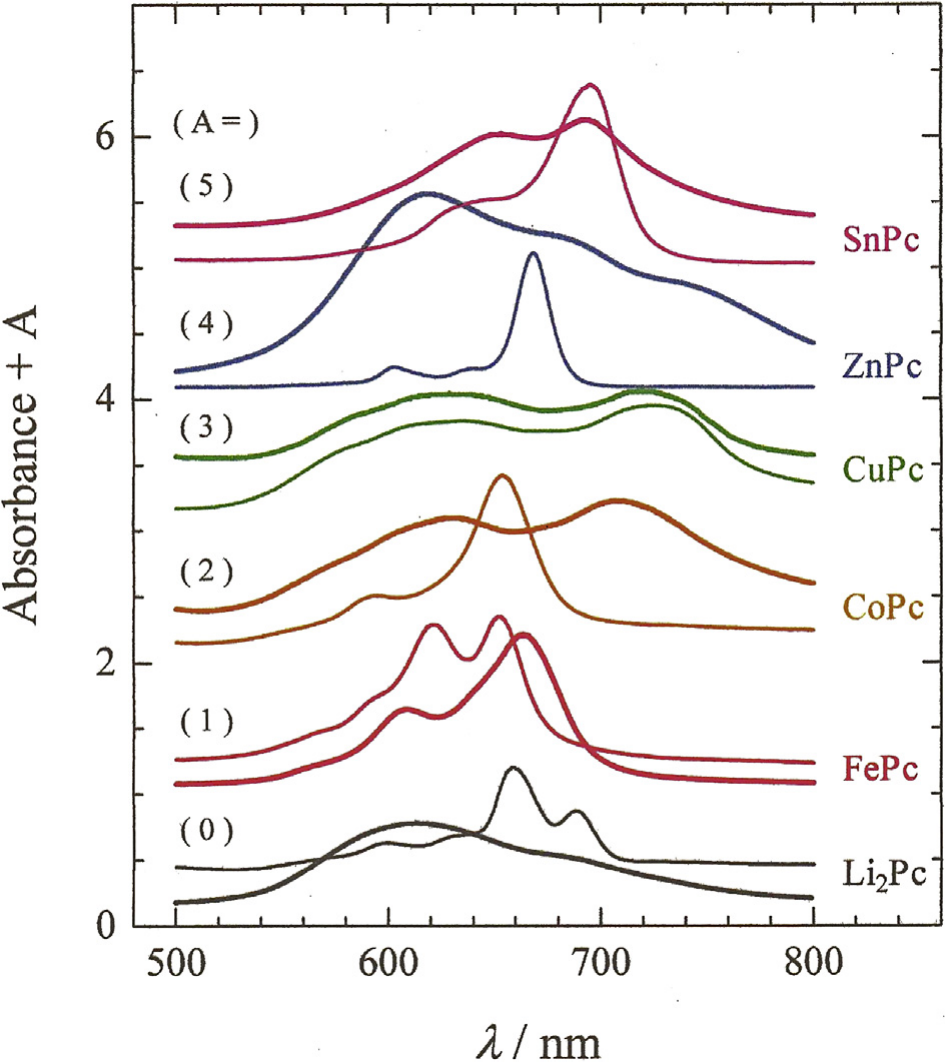
UV–Vis absorption spectra of MPc–polymer complexes in water (with possibly a contamination of organic solvents) and of MPcs in organic solvents. In square brackets, the organic solvent used in Step (1) of the protocol for preparing the water-soluble MPc–polymer complexes is reported. For clarity, the spectra of each MPc series are shifted upwards along the vertical axis direction by a quantity A, where A = 0, 1, 2, 3, 4, or 5. Li_2_Pc: Li_2_Pc–PEG complex in water [DMA],  Li_2_Pc in DMA; FePc: FePc–PVP complex in water [pyridine],  FePc in pyridine; CoPc: CoPc–PEG complex in water [DMSO],  CoPc in DMSO; CuPc: CuPc–PVA complex in water [DMSO],  CuPc in DMSO; ZnPc: ZnPc–PVP complex in water [DMF],  ZnPc in DMF; SnPc: SnPc–PVP complex in water [pyridine],  SnPc in pyridine.

Fig. 2 shows the UV–Vis absorption spectra of different MPc–polymer complexes in water, where MPcs are Li_2_Pc, FePc, CoPc, CuPc, ZnPc, or SnPc, and the polymers are PEG, PVP, or PVA. The figure also shows the spectra of various MPcs dissolved in organic solvents (pyridine, DMSO, DMF, or DMA). In particular, the thin curves are the spectra of MPcs in organic solvents, whereas the thick, bold curves are the spectra of MPc–polymer complexes in water. For clarity, the two spectra of each MPc series are shifted upwards along the vertical axis direction by a quantity of A, where A = 0, 1, 2, 3, 4, or 5.

The UV–Vis spectra of all MPc–polymer complexes in water are characterized by two or three absorption peaks in the 500–800 nm wavelength range. This indicates that each water-soluble MPc (Li_2_Pc, FePc, CoPc, CuPc, ZnPc, or SnPc)–polymer (PEG, PVP, or PVA) complex was prepared.

The peaks due to the complexes Li_2_Pc–PEG, FePc–PVP, CoPc–PEG, ZnPc–PVP, and SnPc–PVP in water were broader and shifted to longer or shorter wavelengths than those due to the corresponding MPcs in organic solvents. These differences are attributed to the following effects: (1) an increase in the degree of stacking of MPc in the complexes as compared with that of the MPc in organic solvent, (2) interactions between MPc and polymer chain, and (3) a change in the solvent of MPcs or MPc–polymer complexes from the organic solvent to water, i.e., solvatochromism, which is the phenomenon observed when the color caused by a solute changes when that particular solute is dissolved in different solvents.

Notably, in Fig. 2, the FePc–PVP complex in water showed the sharpest peak at ∼700 nm among all MPc–polymer complexes. Furthermore, in Step (6), the paste of the FePc–PVP complex was much more largely diluted with water (i.e., more than about 3 times) as compared with that of the other MPc–polymer complexes herein. This experimental result indicates that the intensity of the absorption peak at ∼700 nm for the FePc–PVP complex in water was much larger (i.e., about 3 times larger) than that for the other MPc–polymer complexes in water. Thus, it was concluded that the FePc–PVP complex in water showed the largest and sharpest peak at ∼700 nm among all MPc–polymer complexes.

Generally, the absorption peak at ∼700 nm due to MPc or chlorophyll a in the complexes is assumed to become sharper with the decrease in the degree of stacking of MPc or chlorophyll a [1, 2, 3, 4, 5, 6]. Therefore, the sharpest peak at ∼700 nm for the FePc–PVP complex indicates that the degree of stacking of FePc in the complexes is very small, such as that of a monomer or a similar structure. Conversely, the largest peak at ∼700 nm for the FePc–PVP complex indicates that the FePc–polymer (e.g., PEG, PVP, and dextran) complexes can be easily formed and prepared. These results indicate that FePc–polymer complex may have a relation with the fact that heme in the hemoglobin includes Fe(II). Therefore, we judged that FePc–polymer complexes are suitable for the purpose of our study.

Additionally, we experimentally confirmed that the mentioned absorption peak at ∼700 nm, which is due to the MPc–polymer complex in water, only underwent small changes when the polymer used to prepare it changed.

### 3.2 A suggested mechanism of how the water-soluble MPc–polymer complexes are formed

The MPc–polymer (PEG or PVP) complexes form as a consequence of the hydrophobic interaction between the hydrophobic part of the polymer (–CH_2_–CH_2_– or (–CH_2_–CH–)_n_) and the hydrophobic substance of MPc. On the contrary, the hydrophilic part of the polymer (ether oxygen atoms or pyrrolidone groups) interacts via hydrogen bonds with the water molecules present around the complexes. Consequently, the MPc–polymer complexes dissolve in water. In the case of the water-soluble chlorophyll a–polymer complexes, a similar mechanism can be suggested.

### 3.3 Possibility for the FePc (or chlorophyll a)–polymer complex to break cancer cells

As mentioned above, H. Kobayashi *et al.* [7, 8, 9] succeeded in breaking a cancer cell and a Treg-cell by irradiating IR700–antibody conjugate bound to the surface of these cells with near-infrared light having a wavelength of ∼700 nm. Therefore, speculatively, the FePc (or chlorophyll a)–polymer (e.g., PEG, PVP, and dextran) complex adsorbed onto the surface of a cancer cell may be expected to break the cancer cell via the irradiation of the near-infrared light. Herein, the cancer cell will be directly broken by the heat generated from FePc or chlorophyll a.

Although our group isolated chlorophyll a from fresh spinach leaves [1, 2, 3, 4, 5, 6], pure chlorophyll a can be cheaply (e.g., 130 USD/g) purchased from pharmaceutical companies nowadays (e.g., Fuji SLI Kurorofiru Jigyobu; http://chlo-ken.jp/research/a/). As mentioned in the section of 1. Introduction, the chlorophyll a–polymer complexes can be very easily prepared at room temperature [3, 4]. Additionally, the FePc–polymer complexes can be prepared relatively easily because they showed the largest peak at ∼700 nm in UV–Vis absorption spectra.

The rationale for considering these complexes as a potential tool to break cancer cells is as follows: (1) the degree of stacking of FePc or chlorophyll a in the complex is very small (corresponding to that of a monomer or a similar structure); (2) because FePc or chlorophyll a is wrapped by polymer chains with high biological affinity [10, 11, 12] as well as flexibility, the FePc (or chlorophyll a)–polymer complex may selectively adsorb onto the surface of cancer cells rather than onto the surface of normal cells; herein, the selective adsorption of polymer chains is strongly expected to play an important role; and (3) the complex move slowly *in vivo*; thus, it may remain on the surface of cancer cell for a sufficiently long period to allow it to break the cancer cell via irradiation of near-infrared light.

Recently, polymeric nanostructured materials, e.g., micelles, nanogels, polymersomes, nanoparticles, nanocapsules, nanofibers, dendrimers, and nanocomposites have been studied in detail for the medical applications [13, 14, 15]. Therefore, it is expected that the latest polymer technologies will help the application of FePc (or chlorophyll a)–polymer complex in the cancer therapy in the future.

However, no experiments involving cancer cells and cancer cell lines have been performed at present. Inspections by these experiments are required to be conducted toward the application for cancer therapy. Furthermore, for validation, all complexes must be characterized with further analytical techniques (e.g., fluorescence, NMR, IR, and small-angle X-ray scattering) along with their UV–Vis absorption spectra.

While experimenting with cancer cells and cancer cell lines, if none of the aforementioned complexes can break these cells, more suitable polymers, which contain newly synthesized ones, must be searched to break them [13, 14, 15]. At this time, evaluating the optimum length of polymer chain, i.e., the optimum average molecular weight of the polymer, will become very important. Additionally, the degree of branching of the polymer chain may become very important.

## Declarations

### Author contribution statement

Isamu Inamura: Conceived and designed the experiments; Analyzed and interpreted the data; Wrote the paper.

Kentaro Inamura, Yuji Jinbo: Analyzed and interpreted the data; Wrote the paper.

Toshinao Mihara, Yasuhiro Sasaoka: Performed the experiments.

### Funding statement

This research did not receive any specific grant from funding agencies in the public, commercial, or not-for-profit sectors.

### Competing interest statement

The authors declare no conflict of interest.

### Additional information

No additional information is available for this paper.
